# Unresolvable Pixels Contribute to Character Legibility: Another Reason Why High-Resolution Images Appear Clearer

**DOI:** 10.1177/2041669520981102

**Published:** 2020-12-26

**Authors:** Madoka Ohnishi, Koichi Oda

**Affiliations:** Tokyo Woman’s Christian University, Tokyo, Japan

**Keywords:** legibility, character, letter, high resolution, sample density, spatial frequency, critical band, low vision

## Abstract

This study examined the effect of character sample density on legibility. As the spatial frequency component important for character recognition is said to be 1 to 3 cycles/letter (cpl), six dots in each direction should be sufficient to represent a character; however, some studies have reported that high-density characters are more legible. Considering that these seemingly contradictory findings could be compatible, we analyzed the frequency component of the character stimulus with adjusted sample density and found that the component content of 1 to 3 cpl increased in the high-density character. In the following three psychophysical experiments, high sample density characters tended to have lower contrast thresholds, both for normal and low vision. Furthermore, the contrast threshold with characters of each sample density was predicted from the amplitude of the 1 to 3 cpl component. Thus, while increasing the sample density improves legibility, adding a high frequency is not important in itself. The findings suggest that enhancing the frequency components important for recognizing characters by adding the high-frequency component contributes to making characters more legible.

## Introduction

Are high-resolution characters more legible? How many dots or pixels should be used to present a character? With the development of printing and display technology, various approaches have been used to answer these questions. For example, Bailey et al. (1987) conducted a letter-counting task used a grainy character image with 12 matrices per character height and a smoother image with 24 matrices to render characters on a computer screen, and found that the smooth character image using many matrices was slightly more efficient.

However, it does not seem to be the case that the higher the density, the better the legibility; rather, some studies have reported that there is a critical point in character sample density. Thus, while Erdmann and Neal (1968) reported that the more elements that form a letter, the better the visibility, in most cases, visibility has been found to remain constant once the density exceeds 125 elements per inch. Legge et al. (1985) conducted experiments to measure reading speed for matrix-sampled sentences and blurred sentences and demonstrated that the critical sample density at 1.5° characters was approximately 8 × 8 samples/character, while the critical blur bandwidth was approximately 2 cycles/letter (cpl).

Although characters are broadband stimuli that include various spatial frequency components, it is known that the information of a very limited frequency band (critical band [CB]) greatly contributes to their recognition. Specifically, low-frequency components of approximately 1 to 3 cpl are thought to be important for recognition of single characters (Anderson & Thibos, 2004; Gold et al., 1999; Kwon & Legge, 2011, 2013; Parish & Sperling, 1991; Solomon & Pelli, 1994) and reading (Kwon & Legge, 2012; Legge et al., 1985).

If the spatial frequency component necessary for character recognition is 1 to 3 cpl, from a sampling theory perspective, it should be sufficient to have six dots in each of the vertical and horizontal directions to represent a character. However, recent high-definition displays devote a large number of pixels to displaying one character on screen.

Displaying characters in high density has been thought to improve visibility to some extent (Bailey et al., 1987; Legge et al., 1985). Why are high-density characters easier to read? Is there a critical sample density? We hypothesized that the amplitude of CB would be involved in the effect of character sample density on legibility.

It was reported that the amplitude of relatively low-frequency components (1 to 4 cpl) affected the legibility of characters in different fonts (Arditi et al. (1997). These authors found that the relatively small amplitude of 1 to 4 cpl with the outline font resulted in low legibility. A study using grating stimuli has demonstrated that the square wave gratings had better visibility because the amplitude of the fundamental frequency components is greater in the square wave (Campbell & Robson, 1968). A square wave, unlike a sine wave, contains harmonics that are odd multiples of the fundamental frequency, and these harmonics have an important role in creating edges in the waveform. We assumed that a phenomenon similar to that shown by Campbell and Robson (1968) occurred in characters with different densities.

It was hypothesized that higher density characters, which had finer contours, would be more similar to the square waves in terms of frequency characteristics than lower density characters. It was also hypothesized that high-density characters with square wave-like frequency properties would have bigger CB amplitude, corresponding to the fundamental frequency of square wave grating. The amplitude of CB would relate to the legibility of the characters with different densities. Based on these hypotheses, we performed frequency analysis of the character images and psychophysical experiments.

In this study, contrast thresholds were adopted as a measure of legibility. Legibility is often assessed as a recognition threshold size or reading speed. There were two reasons to measure contrast thresholds in the experiments. The first was that the previous studies mentioned earlier suggested that there would be a dependency of character size on critical sample density. Interestingly, character size and critical sample density were correlated in Legge et al. (1985) and Legge (2007). According to sampling theory, when the blur bandwidth is 2 cpl, the critical sample density should be 4 × 4 samples/character (spl). For small characters, inferences from sampling theory were applied, but for 10° characters, for example, the critical sample density was 20 × 20 spl. Legge (2007) discussed the dependence of critical sample density on the so-called block portrait effect in which the edges of high-frequency components mask low and important spatial frequencies (1–3 cpl) when the character size is large (Harmon & Julesz, 1973). There are also reports that the spatial frequency band important for character recognition depends on character size (Alexander et al., 1994; Chung et al., 2002; Majaj et al., 2002). Oruç and Landy (2009) found that critical sample density may increase due to the shift of CB to high frequency in large characters. However, in these studies, the character size dependency of the frequency channel appeared for large characters of 3° or more. In Legge (2007), the character size and sample density had a linear relationship, so it is difficult to completely explain the size dependence of critical sample density from the size dependence of the CB.

Based on the results of these previous studies, legibility should be measured with constant character size. Although reading speed was an option to avoid the threshold size to measure legibility, we focused to reproduce the findings of Campbell and Robson (1968) in terms of character stimuli, and this was the second reason for our choice. Therefore, legibility in this study was defined as *lower contrast threshold*; in other words, *being able to read even low-contrast text*.

## General Methods

Three experiments were conducted to determine whether an increase in the amplitude of 1 to 3 cpl could affect human perception. The common methods are described first in this section.

**Figure 1.**
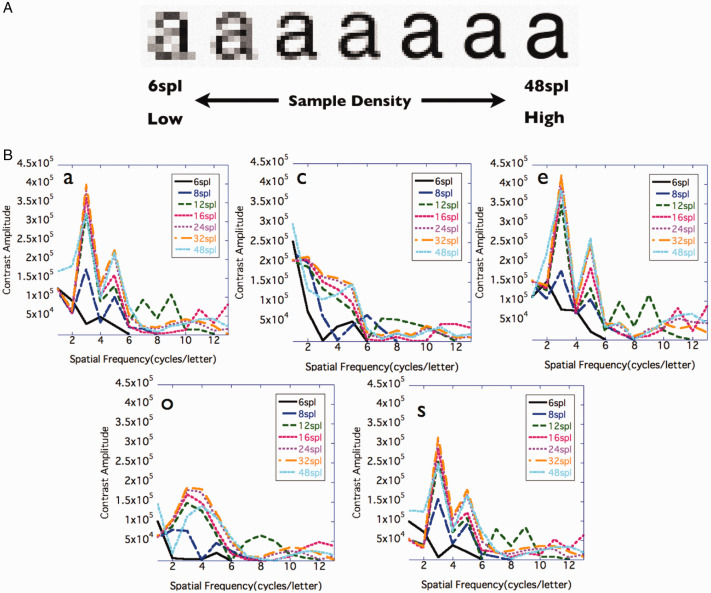
Demonstration of characters with different densities, and difference in spatial frequency characteristics with densities. A: The character sample density (sample per letter; spl) was adjusted. 6 spl means a character image using six samples per character height. B: Relationship between the sample density of the images and spatial frequency components of characters. FFT was conducted by imageJ with the character images (a, c, e, o, and s). From the FFT result of each character image, 0° and 90° power (amplitude = sqrt (spectrum)) were averaged. Those with 0 to 13 cpl were plotted for each sample density.

Before the experiments, fast Fourier transform (FFT) was performed on character images of different sample densities to investigate the frequency characteristics and in particular the amplitude of the CB component (Figure 1).

We adjusted the blocks that make up the character and performed FFT analysis to examine the spatial frequency characteristics of the character images. Since previous research reported no difference in the accuracy of character recognition depending on whether anti-aliasing is performed on character images (Li et al., 2000), in this study, we used a character image that had been subjected to anti-aliasing so that it would be closer to what we use in everyday life. In high-density images, 3 cpl contrast components, which are critical for character recognition, increased. Increasing the density means creating room to add harmonics. Thereby, the edges of the character are prominent, and the character is considered to have a more *square wave-like* frequency characteristic.

From FFT, it was suggested that low-frequency components would be enhanced by adding high-frequency components.

### Stimuli

Five lowercase roman characters which are similar in form (a, c, e, o, and s) were used as stimuli in Experiments 1 and 3. We performed some preliminary experiments to choose the options and found that the thresholds were not stable when some of the choices were prominent. We decided to choose characters that were all similarly round in shape, as none of them stood out and were not easy to recognize. The stimulus characters were rendered in Helvetica Regular font. In Experiment 2, character-like symbols were used as stimuli.

The original images were eight-bit grayscale with a pixel value of 0 for characters or symbols and a background of 255. All images were 96 × 96 pixels. The image sample densities were 6, 8, 12, 16, 24, 32, and 48 samples/letter-height (spl). They were created by sampling from the original images using the area average method.

The character and symbol images were embedded in a base of 125 pixels square with a pixel value of 255. The stimuli were generated by adjusting their Michelson contrast from 0.012 to 0.998 for each sample density condition.

The eight-bit grayscale image has only 256 brightness levels, so the minimum contrast that can be presented is 0.01. White noise was added to the stimuli because this minimum contrast was too high to measure the contrast threshold. Based on the results of the preliminary experiment, white noise with an average pixel value difference of 0 and standard deviation (*SD*) = 12 was added.

The size of the stimuli was set so that the CB component (3 cpl) could be resolved sufficiently and the 9 cpl component, which is the third harmonic of CB, was close to the visual acuity limit and not easily resolved.

### Apparatus

The stimuli were displayed on an iMac 27inch (Desktop computer, CPU2.7 GHz, Intel Core i5, 4 GB 1333 MHz DDR3, AMD Radeon HD 6770M 512 MB), running Mac OS 10.7.4. The resolution of the monitor was 2,560 × 1,440 pixels. The γ value of the display was 2.27. The maximum luminance was 306.53 cd/m^2^, while the minimum was 0.38 cd/m^2^. The maximum luminance contrast was 0.998, while the minimum was 0.01. The experimental session was controlled by a program written in PHP 5.3.28.

### Participants

Participants were recruited for each experiment, although some participated in both Experiments 1 and 2. Participants in Experiments 1 and 2 had normal or corrected-to-normal vision. Participants in Experiment 3 had low vision. There were 17 participants in Experiment 1, 14 in Experiment 2, and 11 in Experiment 3.

The experiments were conducted in accordance with the Code of Ethics of the World Medical Association (Declaration of Helsinki). All participants were informed about the experiment and provided written consent to participate.

### Procedure

Contrast thresholds were measured for each sample density condition using the staircase method. The participants viewed the stimuli at a certain distance by using a chin rest in a dark room. The observation distances are described in the method sections for each experiment. The flow of an experimental session started with the highest luminance contrast. In Experiments 1 and 3, one of the five roman characters was displayed, and in Experiment 2, one of the two symbol images was displayed. When the experimenter hit a key, the experimental program presented the stimulus, and the participant was asked to verbally answer which character or symbol had been displayed.

If the participant answered correctly, the stimulus with a one-step lower contrast was presented. If the answer was incorrect, the stimulus with a two-step higher contrast was presented. It is reported that when staircases have 20 steps or less, accuracy can be improved if the size of a step up was larger than half the spread of the psychometric function (García-Pérez, 1998). As we had prepared a maximum of 20 steps, we decided the number of steps in case of incorrect answers in accordance with García-Pérez (1998).

A five-alternative forced choice (AFC) setup and the one-up/one-down method were used in Experiment 1, so the chance level was 0.2. In Experiment 2, two-AFC and the one-up/two-down method were used. The ratio of correct answers at the threshold was 0.6 and 0.7, respectively.

Fourteen sequences were created so that the same characters and symbols were not displayed more than 3 times consecutively. The display order of characters or symbols was determined using a different sequence for each session.

The presentation of stimuli was repeated until the reversal from correct to incorrect answer or from incorrect to correct answer had been recorded 30 times. The average value of the latter 20 reversals was recorded as the contrast threshold for that session. A total of 14 sessions were performed, 2 sessions for each sample density condition. The average contrast threshold for two sessions was used as the participant’s contrast threshold. The order of the sessions varied randomly for each participant. Each participant completed the experiments within 2 to 3 hours.

### Analysis

In each experiment, one-way analyses of variance (ANOVAs) were conducted to examine the effect of sample density on the contrast threshold. When the sphericity assumption was rejected (*p* < .05), the number of degrees of freedom was corrected using Greenhouse–Geisser’s ε. When significant primary effects were observed, Bonferroni post hoc tests were performed. These analyses examined whether and how the contrast threshold varied depending on the sample density.

We also examined whether the amplitude of specific spatial frequencies in the image affected the contrast threshold.

A square wave contains harmonics that are odd multiples (3 times, 5 times, 7 times, etc.) of the fundamental frequency, and the amplitude of fundamental frequency components is 4/π (approximately 1.28 times) compared with a sine wave. Campbell and Robson (1968) demonstrated in their study that the amplitude of fundamental frequency components affects the contrast threshold of gratings. The difference in contrast threshold due to sample density would be explained from the amplitude of CB components, as in previous research.

We attempted to predict the contrast threshold at each sample density using an average amplitude of CB (1–3 cpl).

(1) ECS=RCB×cs,

where ECS is the estimated contrast sensitivity and RCB is the average value of 1 to3 cpl amplitudes of stimuli (a, c, e, o, and s) at each sample density condition, divided by the amplitude at the highest sample density stimuli. The contrast sensitivity of each participant with the highest sample density stimuli was set as cs.

## Experiment 1: The Effect of Sample Density of Characters on Contrast Threshold With Normal Vision

The purpose of Experiment 1 was to determine the relationship between the sample density of a character image and its contrast threshold. From the FFT, when the character sample density was increased, the CB component amplitude also increased (Figure 1). Studies using gratings (Campbell & Robson, 1968) suggested that the amplitude of the fundamental frequency component affected contrast sensitivity. Therefore, enhancement of the CB component would also affect human character recognition.

In Experiment 1, the contrast threshold was estimated using characters with different sample densities to ascertain if the results were consistent with previous studies (Bailey et al., 1987; Erdmann & Neal, 1968) in which increasing the sample density increased legibility. We also examined whether the contrast threshold could be explained from the amplitude of the CB component. It was predicted that the difference in sample density would be described as the CB component amplitude, and the contrast threshold would be explained from the amplitude of the CB component.

### Stimuli

**Figure 2.**
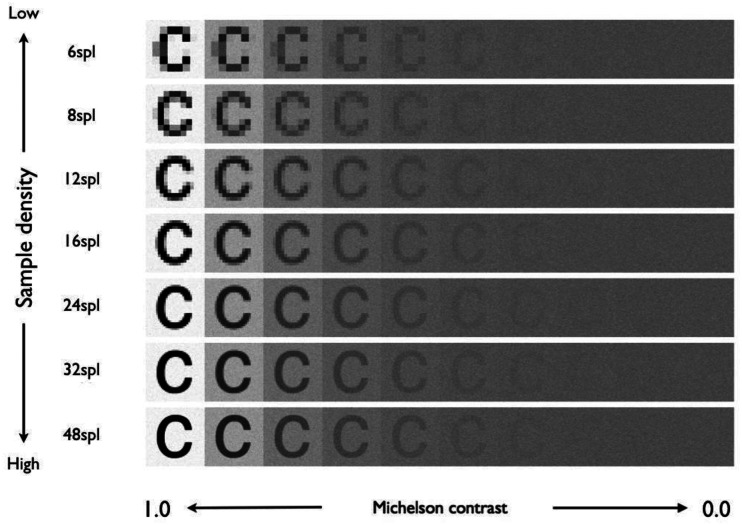
Example of stimuli used in Experiment 1.

As stimuli, eight-bit grayscale images of five lowercase roman characters with similar shapes (a, c, e, o, and s) were used. The original pixel value of the characters was 0, and the background was 255. The Michelson contrast of the stimuli was prepared in 10 × 0.3 log steps from 0.01 to 0.998, and 0.0 (Figure 2). The size of the stimulus was 18.9 minutes of visual angle, with a component of 3 cpl (9.5 cycles/deg) sufficiently resolved for normal vision. The average luminance of the stimulus was 89.85 cd/m^2^ (pixel value 128).

In the range of the stimulus near the threshold value (stimulus using pixel values of 96 to 160), the relationship between the pixel value and luminance was linear. The components of 3 cpl included in the image were the same as in Figure 1, and the contrast component (power) of 3 cpl for 48 spl was 1.14 times that for 6 spl and 1.02 times that for 8 spl (Figure 1).

### Participants and Procedure

Seventeen participants engaged in this experiment. All had normal or corrected-to-normal vision and were native Japanese speakers. Their average age was 24.06 (*SD* = 4.80) years, and their average visual acuity was −0.13 logMAR (*SD* = 0.06).

Participants viewed the stimuli at a distance of 400 cm using a chin rest in a dark room. The contrast threshold was estimated by the staircase method for each sample density condition. Altogether, 15 sessions were conducted, including practice.

### Results and Discussion

**Figure 3.**
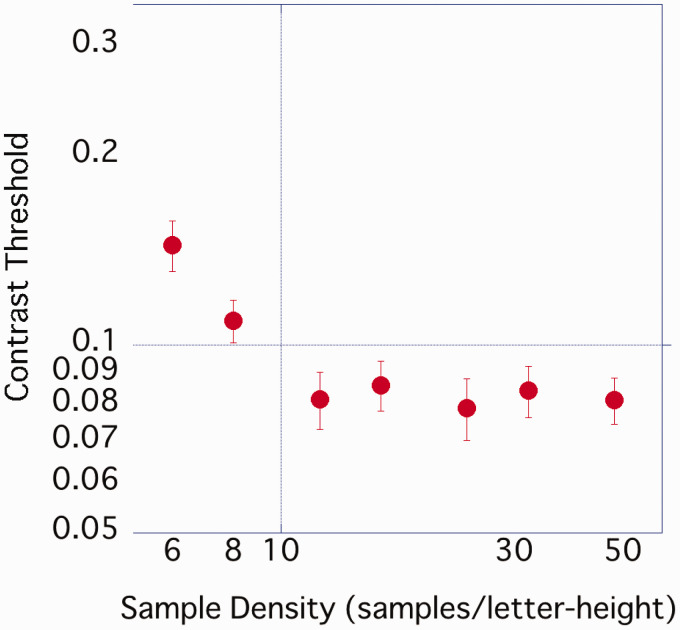
Average contrast threshold of each sample density. Error bar represents standard error.

The average contrast threshold of the 17 participants was estimated (Figure 3) and found to gradually decrease from 6 spl to 12 spl but tended not to differ greatly from 12 spl to 48 spl.

A one-way ANOVA was performed on the obtained contrast threshold with sample density as a factor. A significant main effect of density was observed, *F*(2.84, 45.41) = 30.26, *p* < .001, η_p_^2^ = .654, and multiple comparisons by Bonferroni’s method showed that 6 spl > 8 spl > 12 spl or more (*p*s < .05), with no significant difference at the 5% level under the conditions of 12 spl or more.

Character images with a high sample density tended to have more CB components (Figure 1) and a lower contrast threshold (Figure 3) than those with a low sample density. The result of our experiment was consistent with Campbell and Robson (1968), who reported that the difference in the fundamental frequency component affected the difference in the contrast threshold of the grating stimuli, and Arditi et al. (1997), who suggested that the component amplitude of 1 to 4 cpl explained the contrast threshold of outline fonts. We used Equation 1 to predict the contrast threshold at each sample density for each participant from the amplitude of the CB component (the average value of 1–3 cpl amplitudes).

**Figure 4.**
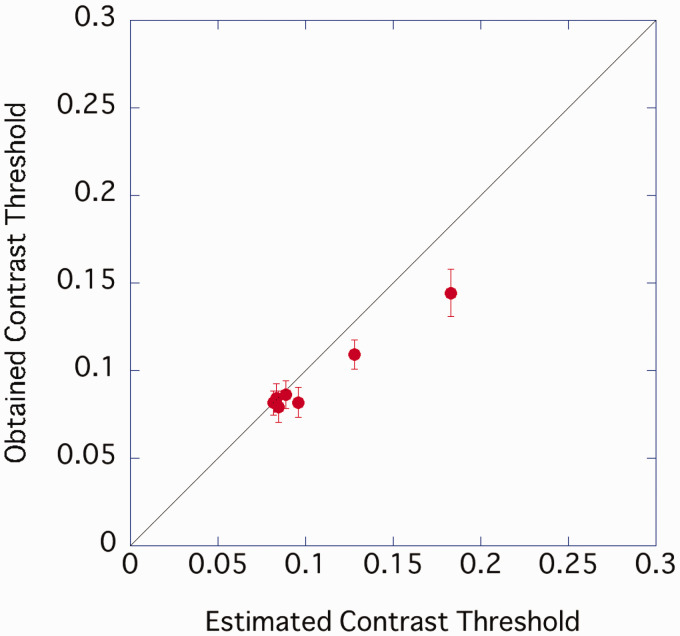
Relationship between predicted contrast threshold and the result of Experiment 1. Error bar represents standard error.

Figure 4 plots the predicted contrast threshold (the reciprocal of the estimated contrast sensitivity) and the average value of the participants’ actual data. The explanation rate of the linear regression equation (intercept 0) for explaining the measured value from the predicted value was *R*^2^ = .828, indicating that the CB component amplitude may have contributed to the contrast threshold of characters with different sample densities. Characters with low sample densities may require higher contrast to read due to the lower CB content. However, the slope of the regression equation was slightly shallow at 0.87, and when the sample density was low, the contrast threshold was lower than the predicted value.

In Experiment 1, the contrast threshold for a character image of 6 to 48 spl was measured. From 12 to 16 spl, increasing the sample density reduced the contrast threshold and made it easier to read. Similar results were obtained as in previous studies (Bailey et al., 1987; Erdmann & Neal, 1968) using character count efficiency and correct answer rate as indices. Participants observed the stimulus from a distance of 400 cm, and it was difficult to resolve high frequencies of 9 cpl or higher. Nevertheless, the result that the contrast threshold was low in a high-density character image suggests that the information content of low-frequency components may have affected perception.

The amplitude of the CB component predicted the contrast threshold of characters with different sample densities. As the amplitude of the CB component increased, the contrast threshold tended to decrease. From Figure 3 and the results of ANOVA, the contrast threshold hardly changed from 12 or 16 spl. This was considered to be related to the fact that the amplitude of the CB component became almost constant after 12 spl.

**Figure 5.**
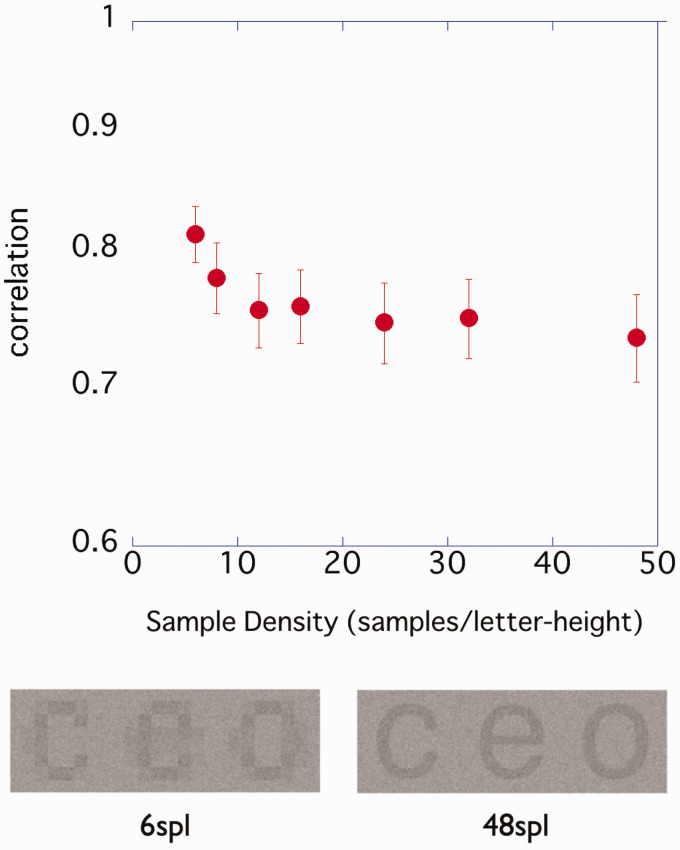
Relationship between sample density and similarity. The upper panel is a plot of the average stimulus correlation coefficient for each sample density (error bar represents standard error). The lower panel is an example of the similarity of stimuli at sample densities of 6 and 48 spl with contrast 1.0.

However, in Experiment 1, the change in sample density was not only related to the amplitude of the CB component. As the sample density decreased, the stimulus letters became more similar (Figure 5).

 According to Kwon & Legge (2013), blurred characters cannot be recognized unless the contrast is high because the perceptual similarity between characters increases as the level of blur increases. Therefore, in Experiment 2, the experiment was performed using stimuli in which the cross correlation between the image resolution and stimulus does not change covariably.

## Experiment 2: The Effect of Sample Density of Character-Like Symbols on Contrast Threshold With Normal Vision

In Experiment 1, the difference in contrast threshold depending on the character sample density was determined by the CB component amplitude. However, there was a covariant relationship between sample density and similarity between the stimulus characters, and it was difficult to consider their effect separately.

Therefore, the same procedure was performed using a symbol image whose similarity does not change depending on the sample density to confirm whether the same result would be obtained as in Experiment 1.

### Stimuli

**Figure 6.**
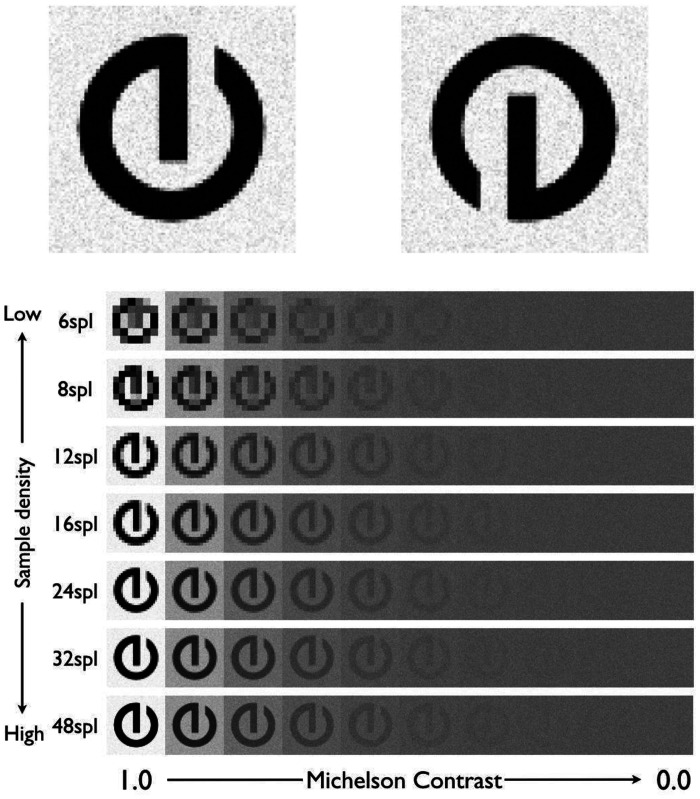
Stimuli used in Experiment 2.

Symbolic stimuli resembling letters were created in which the sample density and similarity of the stimuli did not change (Figure 6).

 Shah et al. (2011) mentioned that the threshold size was larger when there were more options because the characters might be more similar to each other. Although similar, 2 AFCs had significantly larger threshold sizes than the other conditions did, and there was no significant difference in threshold sizes between 4 and 26 AFCs in that study. It cannot be assumed that simply increasing the number of options would have a significant effect on threshold size. Besides, it was difficult to prepare a lot of the letter-like similar patterns. Therefore, two very similar character-like patterns were used in Experiment 2.

Previous research has reported that E is more suitable for estimating the threshold value than C (Reich & Ekabutr, 2002), but since Tumbling-E has many straight lines and is less likely to be anti-aliased, the effect of sample density would differ from Experiment 1. Therefore, symbols whose critical frequency for determining their phase was considered to be 1 to 3 cpl were used in this experiment.

**Figure 7.**
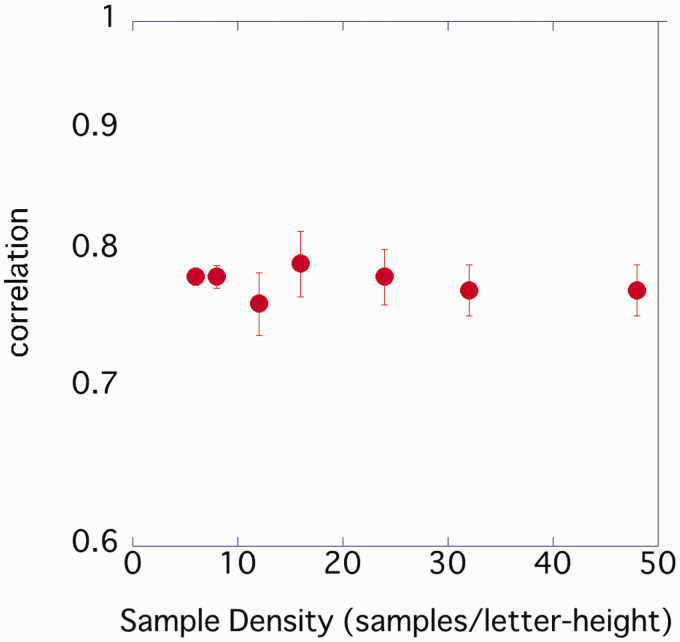
Relationship between sample density and similarity for stimuli (error bar represents standard error).

**Figure 8.**
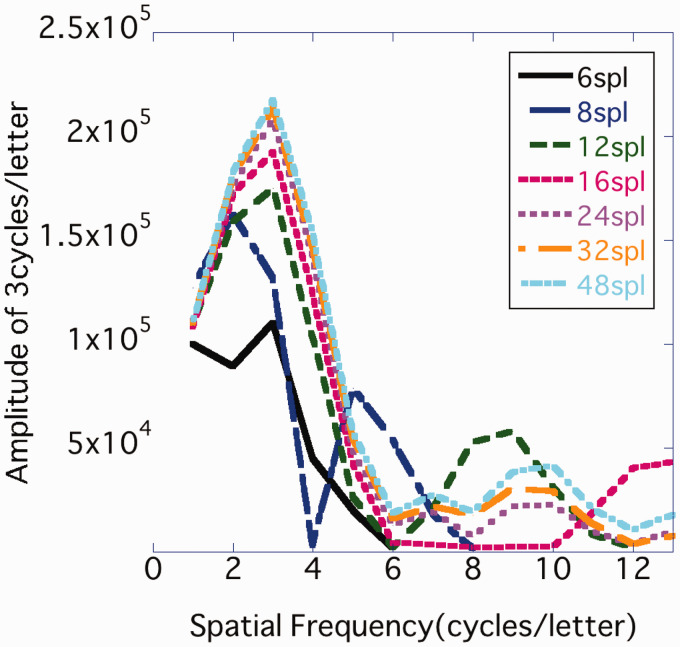
Sample density and spatial frequency components of stimuli in Experiment 2. From the FFT results of each symbol image, 90° power (amplitude = sqrt (spectrum)) of 0 to 13 cpl was averaged for each sample density condition.

Images were created similar to the roman letter e but rotated by 90° and 270°, and sampling and contrast adjustment were performed in the same manner as in Experiment 1 (Figure 6). The cross correlation of the stimulus images was measured for each sample density condition. In Experiment 1, the largest difference in cross correlation was .08 at the maximum, but in Experiment 2 it was .02 (Figure 7); thus, it was considered that the effects of covariation of image resolution and similarity could be controlled as much as possible. The spectrum of each image was as shown in Figure 8, where the contrast component power of 48 spl was 1.11 times that of 6 spl and 1.02 times that of 8 spl.

### Participants and Procedure

Fourteen participants engaged in this experiment. All had normal or corrected-to-normal vision and were native Japanese speakers. Their average age was 22.72 (*SD* = 1.19) years and their average visual acuity was −0.12 logMAR (*SD* = 0.06).

The apparatus and procedure were almost the same as in Experiment 1, but in Experiment 2, there were two types of stimuli, so the contrast step was lowered when the correct answer was given twice in succession.

### Results and Discussion

**Figure 9.**
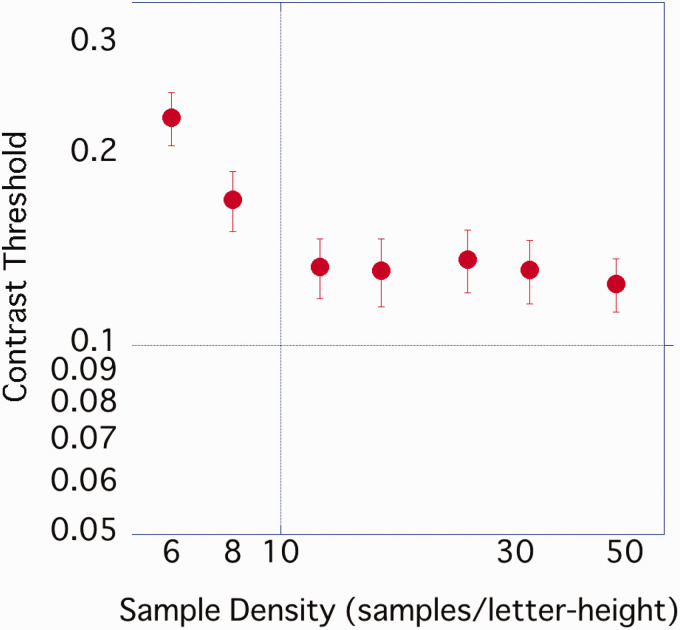
Average contrast threshold of each sample density. Error bar represents standard error.

**Figure 10.**
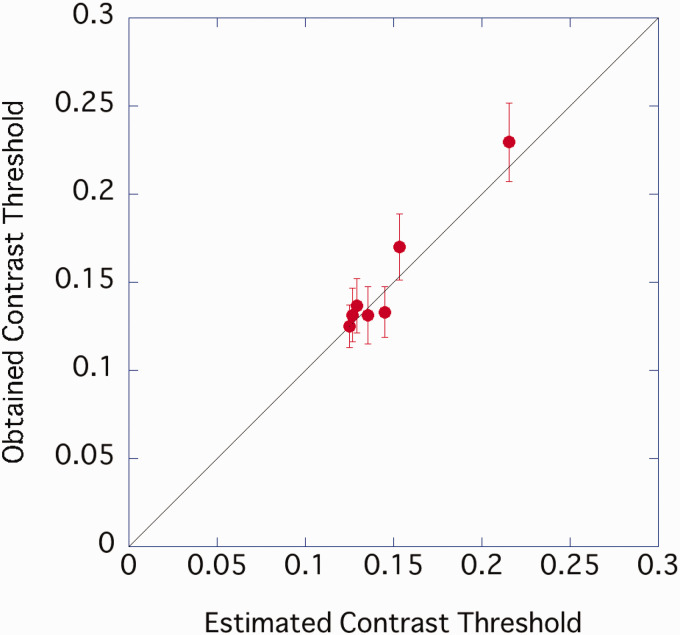
Relationship between predicted contrast threshold and the result of Experiment 2. Error bar represents standard error.

The average value of 14 participants was plotted, with the horizontal axis representing density and the vertical axis representing contrast threshold (Figure 9). As in Experiment 1, the contrast threshold gradually decreased from 6 spl for the grainiest image to 12 spl, but above 12 spl, there was no difference in the contrast threshold even if the density was further increased. One-way ANOVA was performed on the obtained contrast threshold. A significant effect of sample density was observed, *F*(2.34, 30.42) = 18.84, *p* < .001, η_p_^2^ = .592, and post hoc comparison by Bonferroni’s method demonstrated that 6 spl > 8 spl (*p* < .05), 8 spl > 16, 32, 48 spl (*p* < .05). There was no significant difference between the other conditions. The contrast threshold was predicted using expression (Equation 1). The predicted values ​​and measured values ​​were almost the same (Figure 10). The slope of the linear regression equation was 1.03, and the explanatory rate was *R*^2^ = .933.

Experiment 2, in which the similarity between stimuli was controlled using symbolic images, obtained the same results as in Experiment 1. The contrast threshold of symbols with different sample densities was very well predicted from the amplitude of the CB component. The difference between absolute values ​​of the contrast threshold obtained in Experiments 1 and 2 (Figures 3 and 9) would be influenced by participants and the similarity of the stimulus images. As the contrast sensitivity differed for each individual, the absolute value of the contrast threshold may change according to the experiment participant. In addition, symbols similar to e were displayed in Experiment 2, and participants were asked whether the opening was up or down. These stimulus images were very similar (correlation coefficient.77–.79), and thus it was difficult to answer correctly compared with Experiment 1. The result of this experiment was consistent with that of a previous study, which showed that larger thresholds were obtained for two AFCs of letters similar to each other than for four AFCs or more (Shah et al., 2011).

## Experiment 3: The Effect of Sample Density of Characters on Contrast Threshold With Low Vision

In Experiments 1 and 2, it was suggested that high sample density which enhanced the CB amplitude determined the contrast threshold. Although the fine outline was not well resolved, characters with high sample density were more legible due to high CB amplitude.

In Experiment 3, the relationship between the sample density of a character image and its contrast threshold was determined for participants with low vision. It was assumed that a similar trend would be obtained for participants with low vision because the amplitude of CB was more important than having a high-resolution image.

### Stimuli

The same images of roman letters were used as stimuli as in Experiment 1. For Experiment 3, 20 contrast steps (from 0.012 to 0.998, 0.1log step) were used because some participants’ contrast sensitivity was relatively low. Image contrasts were adjusted based on the γ-value of the display.

Stimulus size was determined by the participants’ visual acuity. Stimuli were displayed at twice the size of the letter size at which minimum angle of resolution (MAR) was obtained. It was thought that participants would resolve 3 cycles/letter but would not be able to resolve third harmonics. Stimulus sizes were adjusted by distance from the display.

### Participants and Procedure

Eleven participants engaged in this experiment. They were recruited based on inclusion criteria (Table 1), comprising visual acuity of 0.82 to 1.22 logMAR and 3 times the stimulus size or bigger central field. The presence of contrast sensitivity deterioration, photophobia, or color vision abnormalities was not among the exclusion criteria. Several participants shared their medical records, while for the remaining, we verbally asked about the disease causative of low vision. The apparatus and procedure were the same as in Experiment 1, but the distance ranged from 46 to 116 cm depending on the participant. Visual acuity was measured with Landolt-C before the experiment. It was tested at 30 cm and at each observation distance.

**Table 1.** Profile of Participants for Experiment 3.

Sub.	Age	Sex	Acuity (logMAR)	Distance (cm)	Cause or disorder	Visual field
A	36	F	1.10	61	Monochromat	Normal
B	25	M	1.22	46	Amblyopia	Superior field defect
C	67	F	0.82	114	Congenital cataract	Normal
D	23	M	1.00	76	Retinopathy of prematurity	Normal
E	32	F	1.22	46	Albinism	Normal
F	42	F	1.22	46	Morning glory disc anomaly	Normal
G	39	F	0.89	99	Congenital cataract	Normal
I	39	F	1.10	61	Congenital cataract	Normal
H	51	M	1.10	61	Retinopathy of prematurity	Normal
J	39	M	1.22	46	Congenital cataract	R: defected, L: concentric contraction (15°)
K	34	M	1.10	61	R: Microphthalmia, L: retinal detachment	R: defected, L: normal

*Note.* Cause of disorders as reported by participants. MAR = ■.

### Results and Discussion

**Figure 11.**
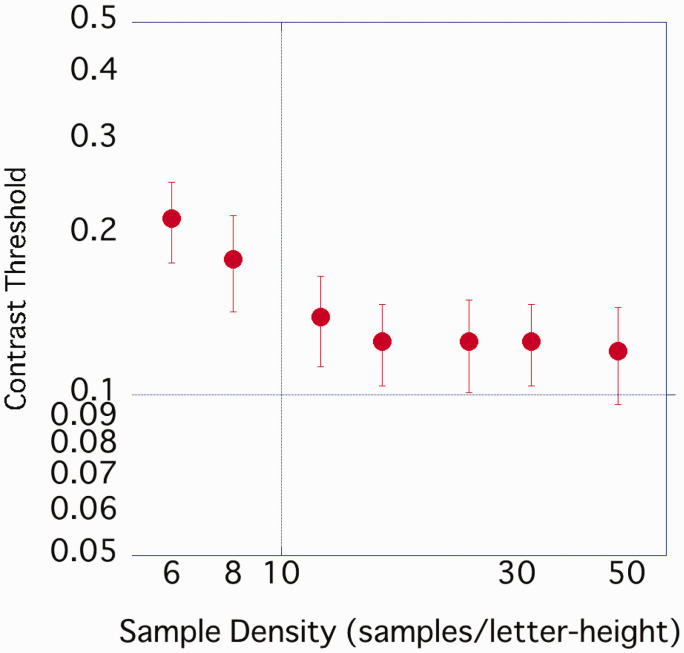
Average contrast threshold of each sample density. Error bar represents standard error.

The average value of the 11 participants was plotted, with the horizontal axis representing density and the vertical axis representing contrast threshold (Figure 11). As in Experiments 1 and 2, the contrast threshold gradually decreased from the coarsest 6 spl image to 12 spl, but above 12 spl, there was no difference in the contrast threshold even if the density was further increased. One-way ANOVA was performed on the obtained contrast threshold. A significant effect of sample density was observed, *F*(2.73, 27.31) = 12.84, *p* < .001, η_p_^2^ = .562, and post hoc comparison by Bonferroni’s method showed that 6 spl > 8 spl (*p* < .05). There was no significant difference between the other conditions.

**Figure 12.**
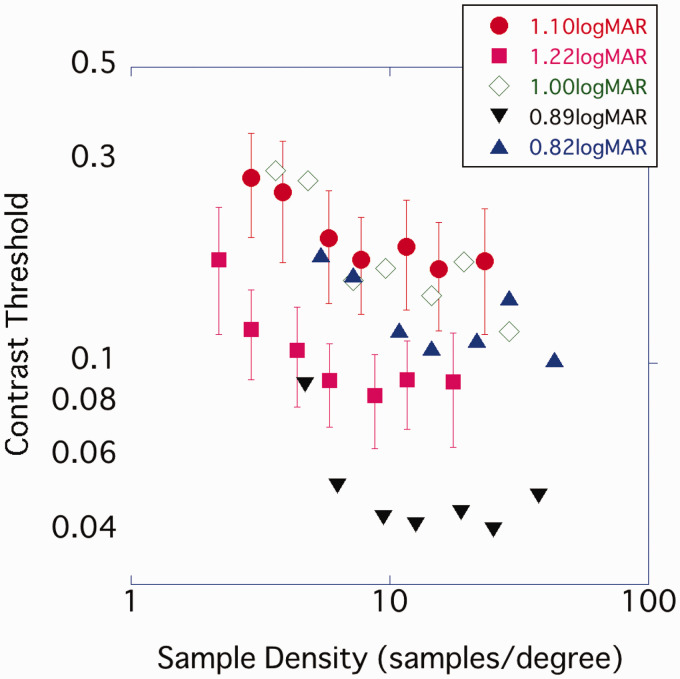
The result of Experiment 3 was replotted for each participant’s visual acuity. The horizontal axis represents the retinal sample density of characters (samples/deg).

**Figure 13.**
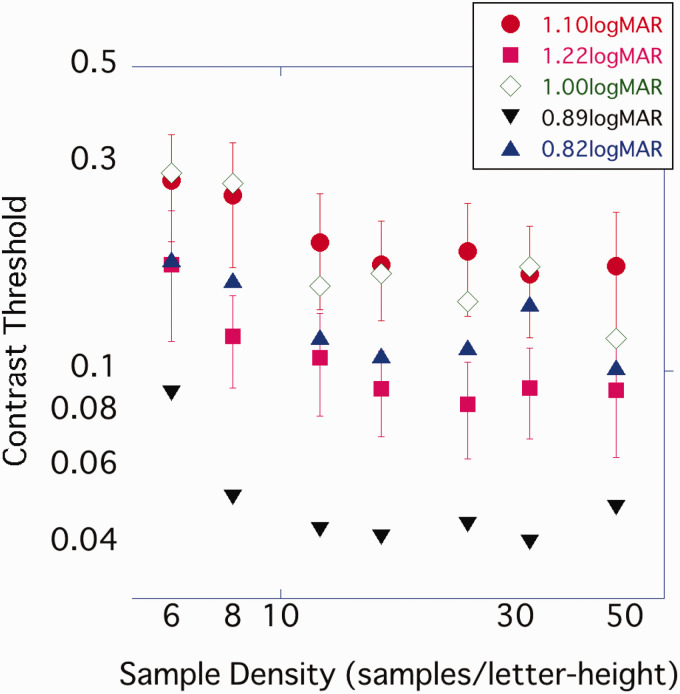
The result of Experiment 3 was replotted for each participant’s visual acuity. The horizontal axis represents the spatial sample density of characters (samples/letter-height).

In Experiment 3, the variance in participants’ visual acuity was greater than in Experiments 1 and 2. The sample density on the retina varied from participant to participant because stimulus size varied with their visual acuity. As a variety of retinal sample densities may have influenced the contrast threshold, results were replotted for each level of visual acuity. When the contrast threshold was plotted with the abscissa plotting the sample density on the retina, the density at which the contrast threshold plateaued differed for each participant’s visual acuity (Figure 12). On the other hand, when plotting the sample density per character height, as in Experiments 1 and 2, the contrast threshold tended to reach its minimum at 12 to 16 spl or above, regardless of the participants’ visual acuity (Figure 13).

**Figure 14.**
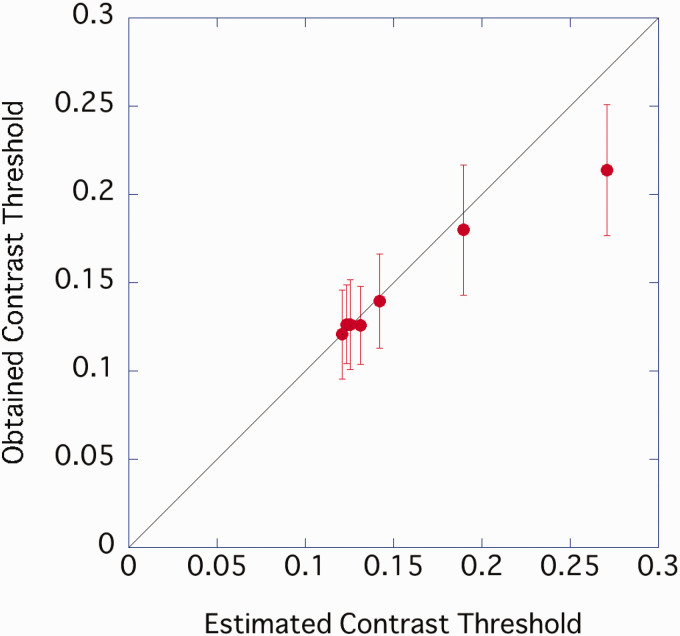
Relationship between predicted contrast threshold and the result of Experiment 3. Error bar represents standard error.

The contrast threshold was predicted using Equation 1. When the contrast threshold was high, that is, when the sample density was low, the measured value had a lower contrast threshold than the predicted value. Otherwise, the predicted and measured values were in good agreement (Figure 14). The slope of the linear regression equation was 0.91, and the explanatory rate was *R*^2^ = .77.

The results for persons with low vision were consistent with those for persons with normal vision. The relationship between sample density and contrast threshold was the same as in Experiments 1 and 2, and the contrast threshold was predicted from the amplitude of the CB component, even when participants could not resolve high frequencies. It was suggested that the enhancement of the CB component caused by the addition of the high-frequency component may have improved the contrast threshold.

## General Discussion

**Figure 15.**
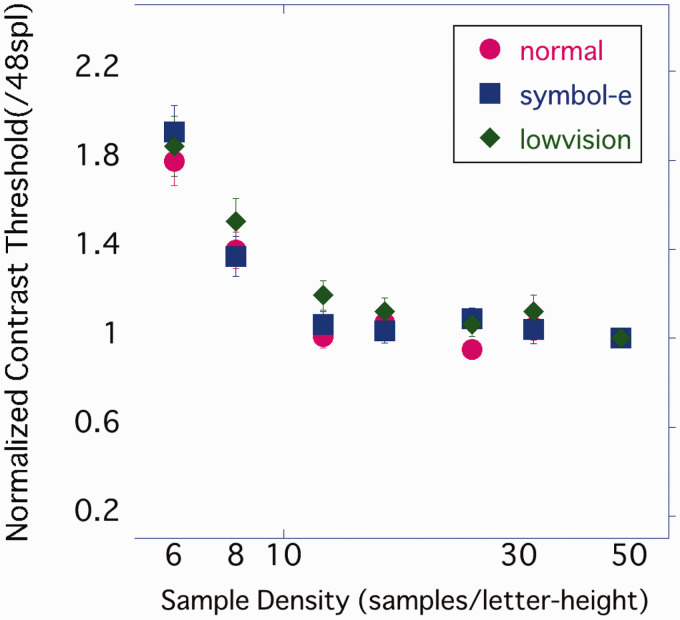
Results of Experiments 1 to 3. Standardized at 48 spl for each individual.

The results of Experiments 1 to 3 suggest that increasing the sample density of characters tends to lower the contrast threshold and make them easier to recognize. However, when the sample density exceeded 12 samples per character height, the contrast threshold no longer decreased and remained almost flat. To confirm whether the results of Experiments 1 to 3 were consistent, their results were compared (Figure 15). The contrast threshold at each sample density was standardized by the contrast threshold at 48 spl for each participant to reduce the effect of individual differences. As a result of the two-way ANOVA using the sample density and experiment as factors, only the main effect of the sample density was significant, *F*(3.24, 126.22) = 79.12, *p* < .001, η_p_^2^ =.67. The main effect of the experiment and interaction were not significant, *F*(2, 39) = 0.972, *p* > .05, η_p_^2^ =.047; *F*(6.47, 126.22) = 0.71, *p* > .05, η_p_^2^ = .035. The averaged contrast threshold was 6 spl > 8 spl > 12 spl or more (*p*s < .001), and there was no significant difference between the other conditions. As the sample density increased, the contrast threshold significantly decreased but did not change after 12 spl. This relationship between the sample density and contrast threshold was reproducible even if the similarity between stimuli was controlled and the experiment performed on participants with low vision who could not resolve high spatial frequency components.

We assumed that the increased CB amplitude would contribute to make characters more legible. Displaying an image at a high density allows a margin to include a high-frequency component, which increases the CB component amplitude of the characters. In the experiment stimuli, characters with a sample density of 12 spl or more contained more CB components than those with lower density (Figures 1 and 8). In addition, in all experiments conducted, participants observed the stimuli from a distance at which high-frequency components of 9 cpl or more were near the resolution limit. This suggests that what contributed to legibility was not the high-frequency component itself that was contained in the image, but the increase in the CB component amplitude due to the addition of the high-frequency component.

Our study results are consistent with those of Bailey et al. (1987), published before image processing using current anti-aliasing was widely performed. The sample densities of the character stimuli used in Bailey’s experiment were 12 and 24 per uppercase letter-height. The lower case letters are approximately half the size, and the sample densities are estimated to be approximately 6 spl and 12 spl, respectively. Although Bailey et al.’s method differs entirely from this study, the reading efficiency of 12 spl characters was slightly better than that of 6 spl characters due to the increased CB content.

Campbell and Robson (1968) measured the contrast sensitivity of grating stimuli and reported that square waves had higher contrast sensitivity because the amplitude of the fundamental frequency was greater than in sine waves. The findings of grating stimuli that increase the amplitude of frequency band, which is important for recognition, affect the contrast threshold that is also applied to the more complex patterns such as characters and symbols. Our findings that the contrast threshold predicted from the ratio of the amplitude of the CB component matched the actual data to some extent were consistent with Campbell and Robson’s finding that the ratio of the amplitude of the fundamental frequency component matched the ratio of the contrast sensitivity of the grating stimuli.

We used roman letters in our experiments, but the findings of this study would also be applicable to letters that share structural similarity with roman letters. However, for more complex characters like kanji, the CB itself would be different (Majaj et al., 2002), so that it might be necessary to have higher resolution for CB component enhancement.

Although the amplitude of the 1 to 4 cpl frequency was integrated to estimate the legibility in Arditi et al. (1997), the averaged amplitude of 1 to 3 cpl, considered as the CB in previous studies (Anderson & Thibos, 2004; Gold et al., 1999; Kwon & Legge, 2011, 2013; Parish & Sperling, 1991; Solomon & Pelli, 1994) was used in this study. The maximum value or the area of the CB may be considered as predictors; however, the components that most affect legibility were not included in the discussion because this was outside the scope of this study. This should be considered in conjunction with the measurement of the CB itself, as in Fiset et al. (2008), who examined letter recognition in terms of phase and frequency.

 It was pointed out by Campbell and Gubisch (1966) that the optical filter may blur high-frequency components. In Experiments 1 and 2 of this study, 3 cpl corresponded on the retina to a frequency of 9.5 cpd, whose contrast attenuation was inferior to that reported by Campbell and Gubisch (1966). In contrast, the retinal frequency corresponding to 9 cpl was 28.6 cpd, whose contrast sensitivity was near zero. The purpose of this study was to investigate the effect of the low-frequency component enhancement on contrast threshold so the experiments were conducted with a setting in which the third harmonics were hardly resolved. Further investigations will be needed to determine how the presence of harmonic components affects legibility in larger letters whose harmonics are also resolvable.

Our results are also related to those of Legge (2007), who found that the critical density varied with character size. The stimulus sizes used in this study ranged from 0.3° to 2.7°, which is similar to the size of the characters used in everyday life. For example, the size of a 9-pt character from a distance of 30 cm is about 0.6° visual angular. However, in some cases, such as when there is a deficit in central vision, it may be necessary to read using large letters. In such cases, the findings of this study may not be applicable, since a previous study has suggested that the critical sample density is dependent on character size (Legge, 2007). A critical sample density of 6.6 samples/letter-height was reported for a character size of 0.25° and 9.5 samples/letter-height for a character size of 2.7°. Since in Legge (2007) reading speed is used as index, it is difficult to compare directly their results with our study. However, in Experiment 3, the results tended to differ from those in Legge. In this experiment, some participants observed a large stimulus with a visual angle of approximately 2°, but the contrast threshold did not change beyond 12 to 16 spl (Figure 11). It was suggested that the sample density of characters rather than the sample density on the retina influenced the contrast threshold (Figures 12 and 13). However, as the number of participants who observed at large font size was too small to predicate the effect of systematic change of the size on the contrast threshold, further consideration is necessary to determine the size effect demonstrated in Legge (2007). Some of the previous studies reported that larger letters had higher CB (e.g., Alexander et al., 1994; Majaj et al., 2002). Majaj et al. (2002) considered the usage of edge information which was reported in square wave gratings (Campbell et al., 1971) that increased the CB of characters. With larger characters, the effect of density might be different from that reported in this study because the components of higher frequency may contribute more to their legibility.

## Conclusion

In this study, we examined the effect of character sample density on the contrast threshold. In all three experiments, the contrast threshold decreased and legibility tended to increase with increasing sample density up to 12 to 16 spl, but not at higher sample densities. The amplitude of the CB component increased up to 12 to 16 spl but remained almost constant under higher sample density conditions. This means that the contrast threshold became constant where the CB component amplitude became constant.

The spatial frequency, which is 3 times the CB in the character image used in this study (9 cpl), was almost the resolution limit on the retinal spatial frequency, and the contrast sensitivity was close to 0. The effect of this third harmonic on the threshold was thought to be very limited. In addition, the contrast threshold predicted from the CB amplitude corroborated the experimental results. Considering these results, it might be unimportant to add a high frequency itself, but the amplitude of frequency components that are important for character recognition should be increased to lower the contrast threshold and make the character easier to recognize. These results were almost equal for participants with normal and low vision, thus demonstrating the potential benefit of providing text information with high density even for low-vision observers who cannot resolve high frequencies.
